# Bacteroides fragilis Empyema Secondary to Ruptured Pyogenic Liver Abscess: A Rare Cause of Septic Shock

**DOI:** 10.7759/cureus.116438

**Published:** 2026-09-17

**Authors:** Mohammad Alali, Tesneem Al-Mwajeh, Michael Talaat, Ahmed Kunwer Naveed

**Affiliations:** 1 Internal Medicine, Henry Ford Macomb Hospital, Macomb, USA

**Keywords:** anaerobic infection, bacteroides fragilis, empyema, hepatic abscess, pleural infection, septic shock (ss), vats decortication

## Abstract

Pyogenic liver abscess is an uncommon but potentially life-threatening intra-abdominal infection associated with significant morbidity and mortality. Although hepatic abscesses most commonly present with systemic infection or intra-abdominal complications, transdiaphragmatic spread into the pleural cavity resulting in empyema is rare. *Bacteroides fragilis*, an anaerobic gram-negative bacillus that forms part of the normal gastrointestinal flora, is a well-recognized pathogen in polymicrobial intra-abdominal infections but is infrequently reported as a primary pathogen in empyema originating from hepatic sources.

We present the case of a 59-year-old man with uncontrolled hypertension, type 2 diabetes mellitus, and alcohol use disorder who presented with acute right-sided chest pain radiating to the back accompanied by dyspnea, fever, nausea, and malaise. On presentation, he was febrile, hypotensive, and tachypneic. Computed tomography (CT) imaging obtained to evaluate possible aortic pathology revealed a mild ascending thoracic aortic aneurysm measuring 4.1 cm without evidence of dissection, as well as a large fluid collection measuring 9.05 × 4.37 cm posterior to the right hepatic lobe concerning for a ruptured hepatic abscess and a moderate right-sided pleural effusion. Abdominal ultrasound confirmed a 9.6 cm hepatic abscess. The patient underwent interventional radiology-guided percutaneous drainage of the hepatic abscess and thoracentesis. Pleural fluid cultures grew *Bacteroides fragilis* and *Staphylococcus warneri*, while culture of the hepatic abscess also yielded *Bacteroides fragilis*, supporting transdiaphragmatic spread of infection from the ruptured hepatic abscess. Despite initial management with broad-spectrum intravenous antibiotics, persistent loculated pleural infection required video-assisted thoracoscopic surgery (VATS) with decortication. The patient subsequently improved clinically and was discharged on oral antibiotics to complete therapy.

This case highlights the importance of early recognition of intra-abdominal sources in patients presenting with empyema and underscores the critical role of prompt diagnostic imaging, targeted antimicrobial therapy, and multidisciplinary source control, including percutaneous drainage and surgical intervention, in achieving favorable clinical outcomes and preventing disease progression.

## Introduction

Pyogenic liver abscess (PLA) is an uncommon but serious intra-abdominal infection associated with considerable morbidity and mortality despite advances in diagnostic imaging and antimicrobial therapy. The incidence of PLA has been estimated at approximately 2-3 cases per 100,000 individuals annually in Western countries [1]. Historically, most cases were associated with biliary tract infections or portal venous bacteremia originating from intra-abdominal infections. Improvements in imaging modalities and interventional radiology have significantly improved early detection and management of this condition [2].

The microbiology of PLA typically reflects gastrointestinal flora and is frequently polymicrobial. Common organisms include *Escherichia coli*, *Klebsiella pneumoniae*, streptococcal species, and anaerobic organisms such as *Bacteroides fragilis* [3]. Among these organisms, *Bacteroides fragilis* is notable for its virulence factors, including capsular polysaccharides that facilitate abscess formation and resistance to host immune responses [4]. Additionally, many strains produce β-lactamase enzymes that confer resistance to several antimicrobial agents, making appropriate antibiotic selection essential.

Although most liver abscesses remain confined to the abdominal cavity, complications can occur when infection spreads beyond the liver. These complications include bacteremia, peritonitis, septic shock, and rarely transdiaphragmatic extension into the pleural space resulting in empyema [5]. When this occurs, bacteria may migrate through direct diaphragmatic erosion, lymphatic spread, or fistulous communication between the hepatic abscess and pleural cavity.

Empyema secondary to hepatic abscess is uncommon but clinically significant because delayed recognition can lead to rapid clinical deterioration. Prompt diagnosis requires careful correlation of imaging findings, microbiologic data, and clinical presentation. To our knowledge, cases of empyema caused by *Bacteroides fragilis* secondary to rupture of a hepatic abscess remain rare in the literature. We present a rare case of *Bacteroides fragilis* empyema secondary to a ruptured hepatic abscess requiring multidisciplinary management, including percutaneous drainage and video-assisted thoracoscopic surgery (VATS). This work was previously presented as a poster at the American College of Gastroenterology Annual Scientific Meeting, Phoenix, Arizona, on October 27, 2025.

## Case presentation

A 59-year-old man with a history of uncontrolled hypertension, type 2 diabetes mellitus, and alcohol use disorder presented to the emergency department with acute right-sided chest pain radiating to the back, accompanied by shortness of breath, fever, and nausea. The patient reported progressive worsening of symptoms over several days prior to presentation. On arrival, he was febrile with a temperature of 38.5°C, hypotensive with a systolic blood pressure of 88 mmHg, tachycardic, and tachypneic. Physical examination revealed decreased breath sounds over the right lung base and tenderness in the right upper quadrant of the abdomen. An initial chest radiograph demonstrated a right-sided pleural effusion with associated right basilar opacity (Figure 1). Initial laboratory evaluation demonstrated leukocytosis with neutrophilia, mild anemia, hyponatremia, and elevated lactate consistent with systemic infection. Renal function tests also revealed mild acute kidney injury. Key laboratory findings are summarized in Table 1.

**Figure 1:**
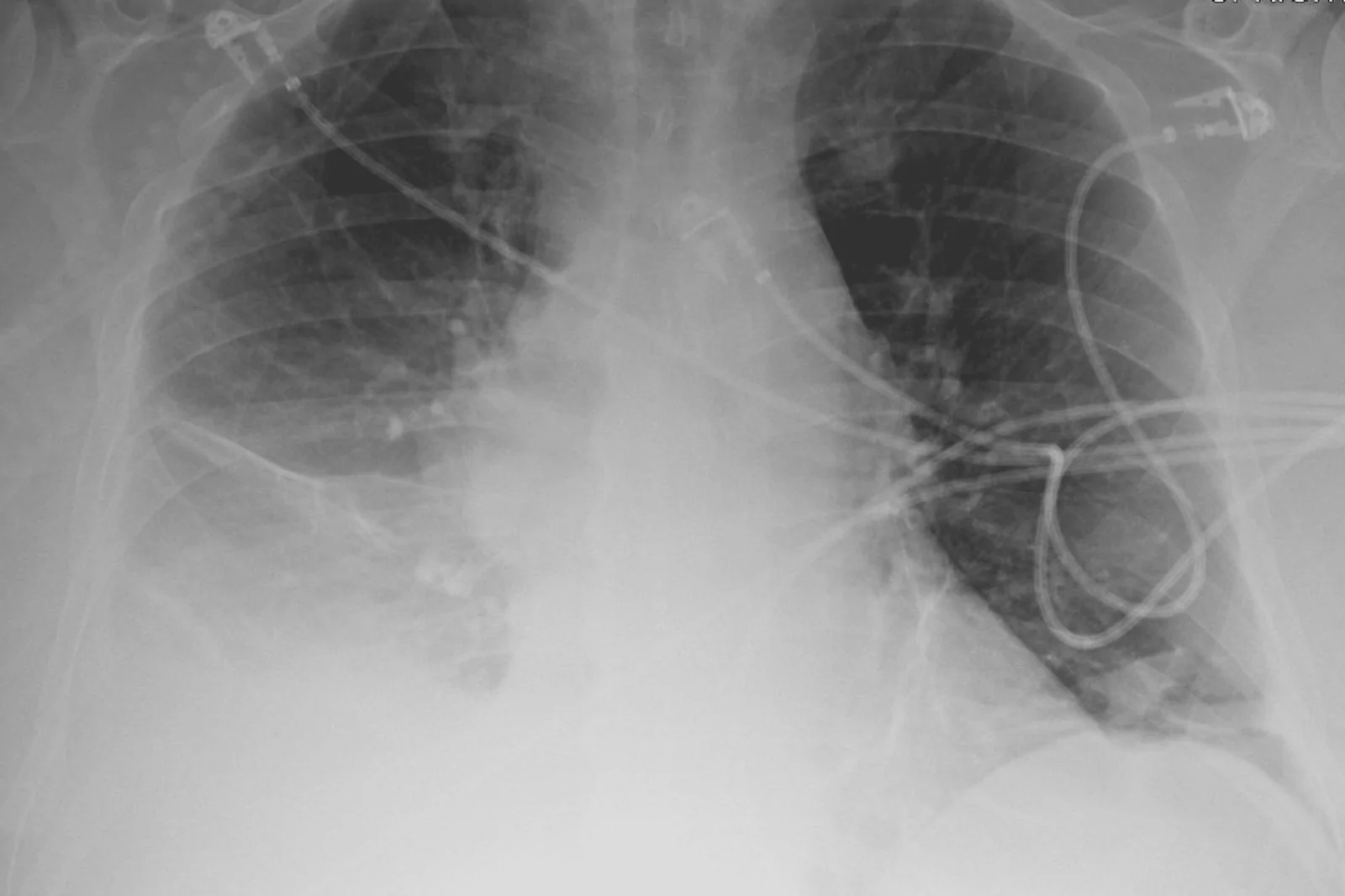
Initial chest radiograph Initial chest radiograph demonstrating a right-sided pleural effusion with associated right basilar opacity.

**Table 1:** Initial laboratory findings on admission Laboratory values obtained at presentation demonstrating leukocytosis with neutrophilia, mild anemia, hyponatremia, elevated lactate, and mild renal dysfunction consistent with systemic infection. Reference ranges correspond to the institutional laboratory standards.

Laboratory test	Result	Reference range
White blood cell count	12.4 K/uL	3.8-10.6 K/uL
Absolute neutrophil count	8.9 K/uL	1.8-7.7 K/uL
Hemoglobin	11.7 g/dL	13.5-17.0 g/dL
Creatinine	1.27 mg/dL	<1.13 mg/dL
Blood urea nitrogen	34 mg/dL	10-25 mg/dL
Sodium	128 mmol/L	135-145 mmol/L
Plasma lactate	2.7 mmol/L	<2.1 mmol/L

Given the severity of chest pain and hemodynamic instability, a contrast-enhanced computed tomography (CT) scan of the chest and abdomen was obtained to evaluate for possible aortic pathology. Imaging revealed a mild ascending thoracic aortic aneurysm measuring 4.1 cm without evidence of dissection. In addition, a large elliptical fluid collection measuring 9.05 × 4.37 cm with thick walls and surrounding inflammatory infiltration was identified posterior to the right hepatic lobe, raising concern for a ruptured hepatic abscess (Figure 2). Imaging also demonstrated a moderate right-sided pleural effusion suggestive of pleural involvement from the intra-abdominal infection (Figure 3).

**Figure 2:**
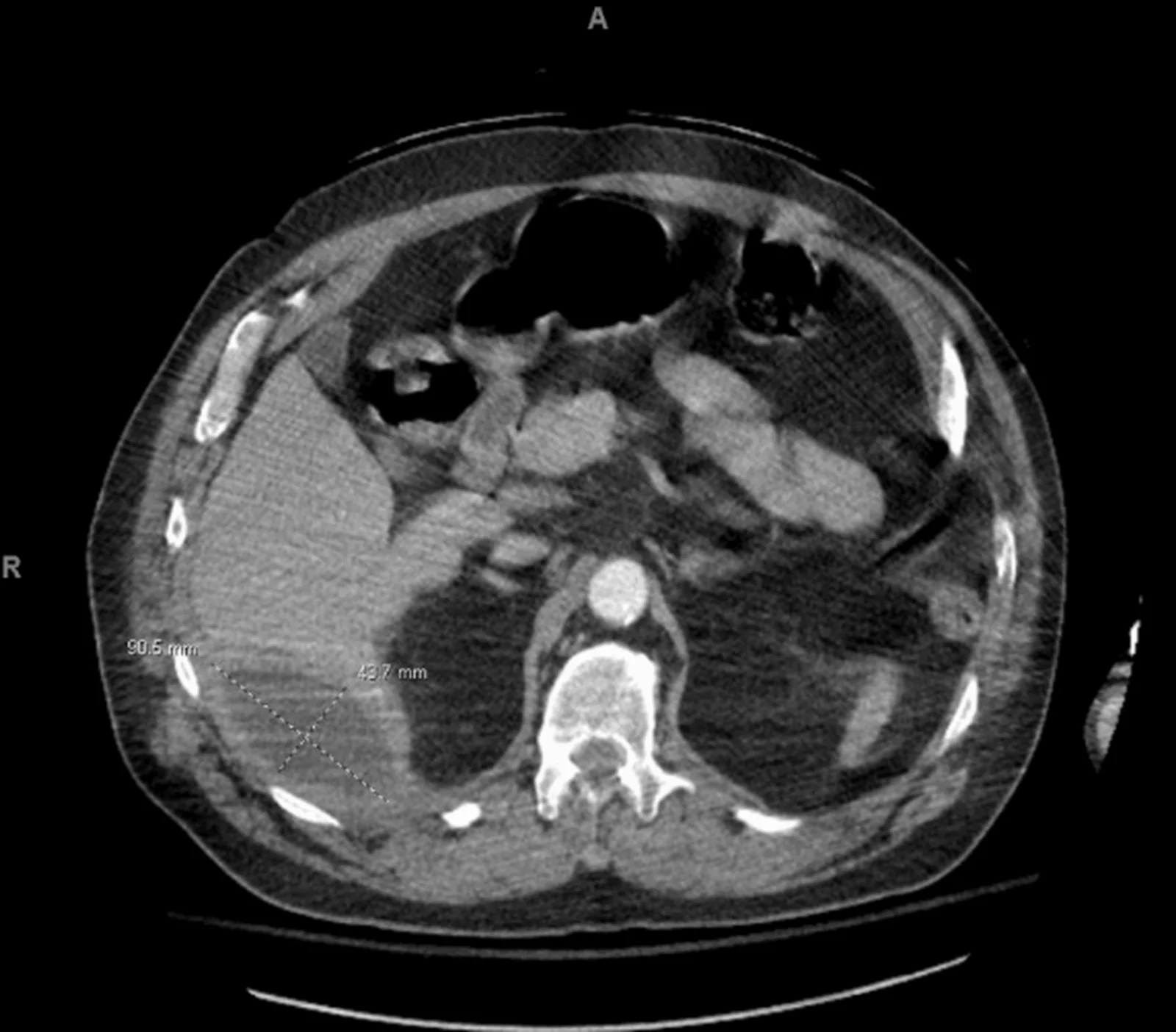
CT of the abdomen demonstrating ruptured right hepatic abscess Contrast-enhanced CT scan of the abdomen demonstrating a large fluid collection in the right hepatic lobe with surrounding inflammatory changes and mass effect on adjacent hepatic parenchyma. Findings are consistent with a ruptured pyogenic liver abscess. CT: computed tomography

**Figure 3:**
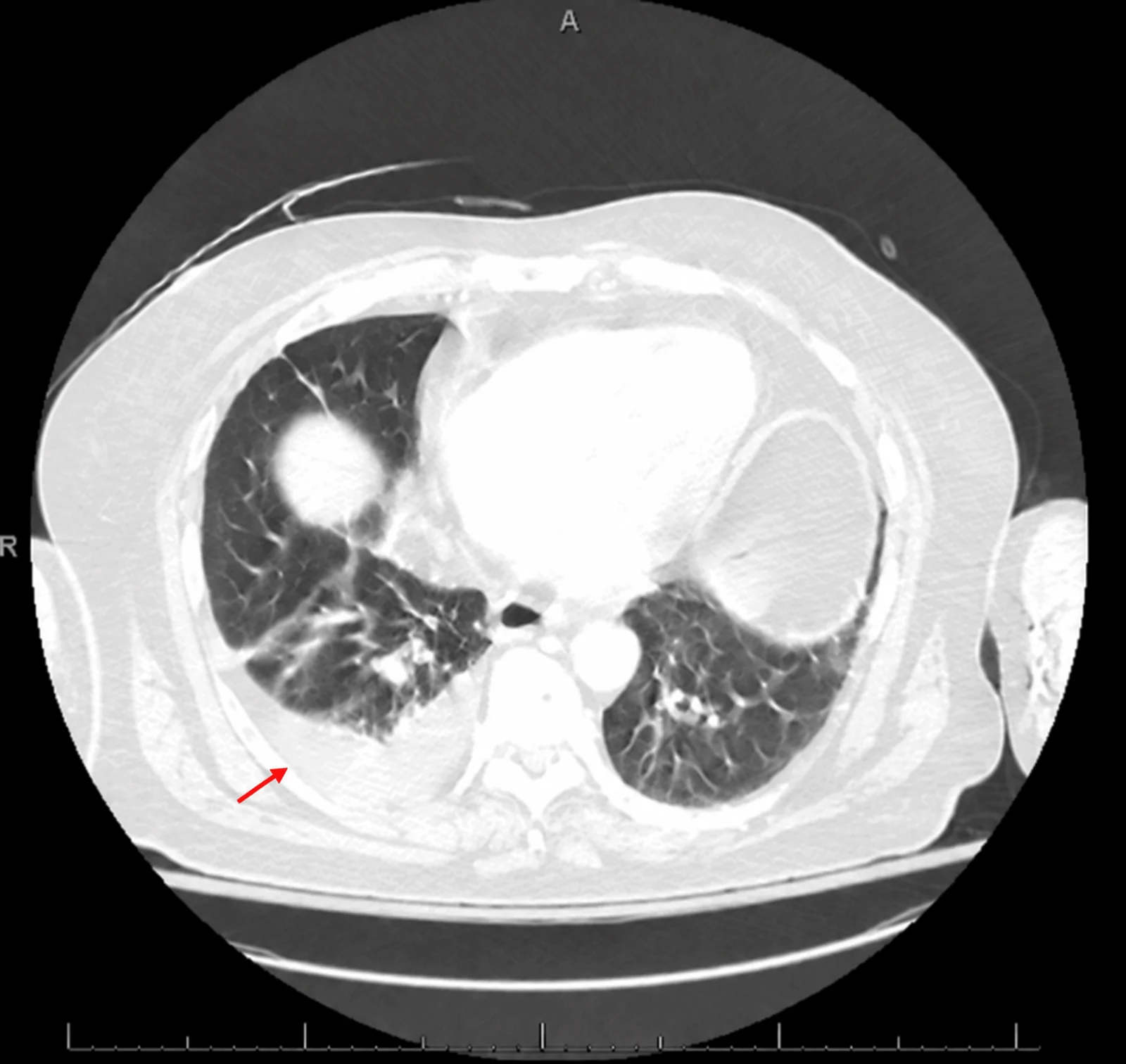
CT of the chest demonstrating right-sided pleural effusion Axial CT image of the chest demonstrating a moderate right-sided pleural effusion with associated compressive atelectasis. The pleural fluid collection is consistent with empyema secondary to transdiaphragmatic spread of infection from the hepatic abscess. CT: computed tomography

Shortly after admission, the patient underwent diagnostic and therapeutic thoracentesis for the right-sided pleural effusion, during which approximately 1 liter of pleural fluid was removed. Following the procedure, the patient developed worsening respiratory distress requiring noninvasive ventilatory support with bilevel positive airway pressure (BiPAP). A planned interventional radiology-guided hepatic abscess drainage procedure was initially attempted but had to be aborted due to the patient’s respiratory instability. A repeat chest radiograph obtained at that time demonstrated interval improvement in the right pleural effusion following thoracentesis (Figure 4).

**Figure 4:**
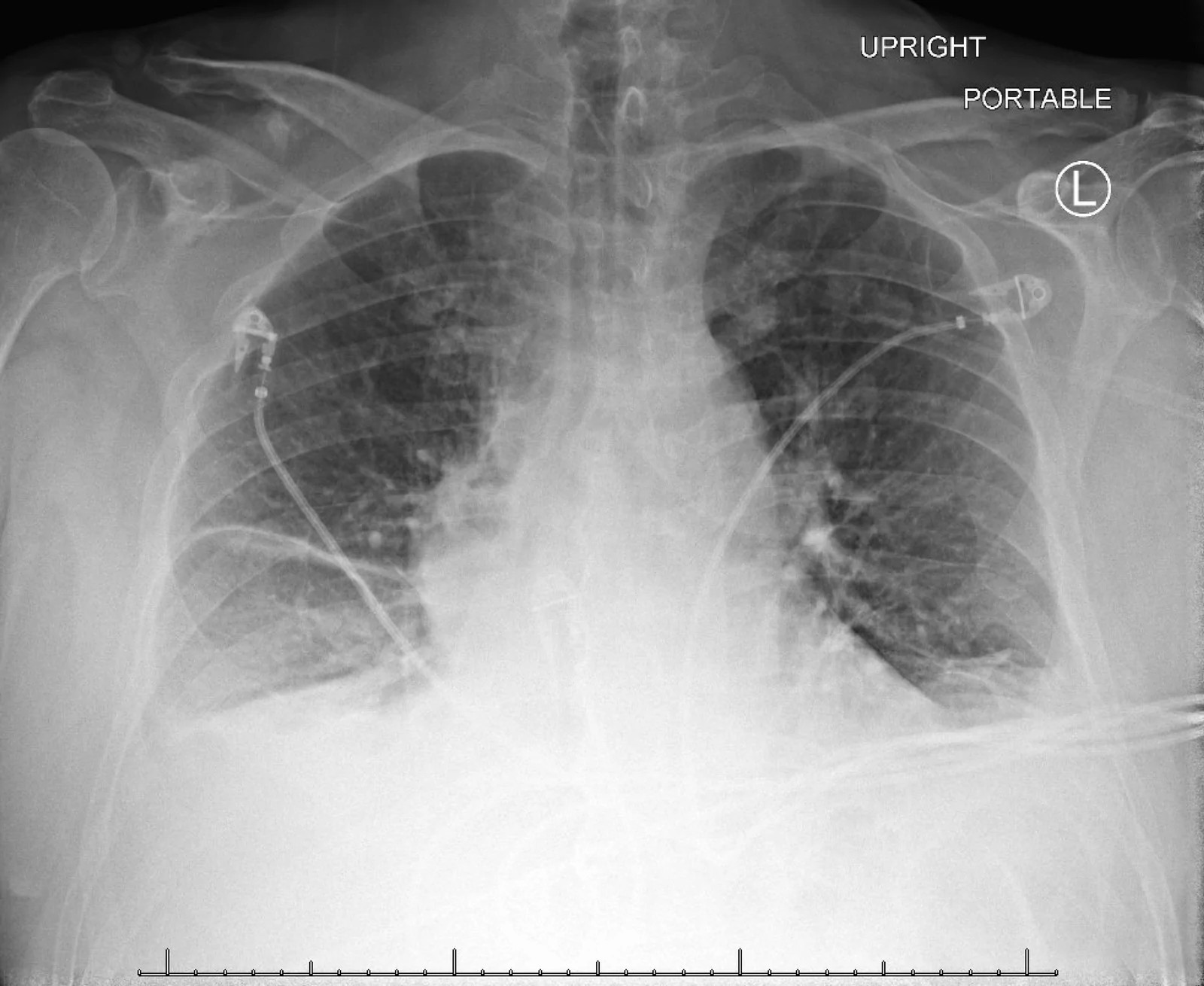
Chest radiograph demonstrating interval improvement in right pleural effusion following thoracentesis Portable upright chest radiograph obtained after therapeutic thoracentesis demonstrating interval improvement of the right-sided pleural effusion with improved aeration of the right lower lung. These findings are consistent with partial resolution of the pleural fluid collection following drainage.

The following day, after stabilization of his respiratory status, the patient successfully underwent interventional radiology-guided percutaneous drainage of the hepatic abscess with placement of a pigtail catheter within the abscess cavity. Subsequent imaging demonstrated appropriate positioning of the drainage catheter with interval reduction in the size of the abscess cavity (Figure 5). The catheter remained in place for several days and was subsequently removed after drainage output significantly decreased.

**Figure 5:**
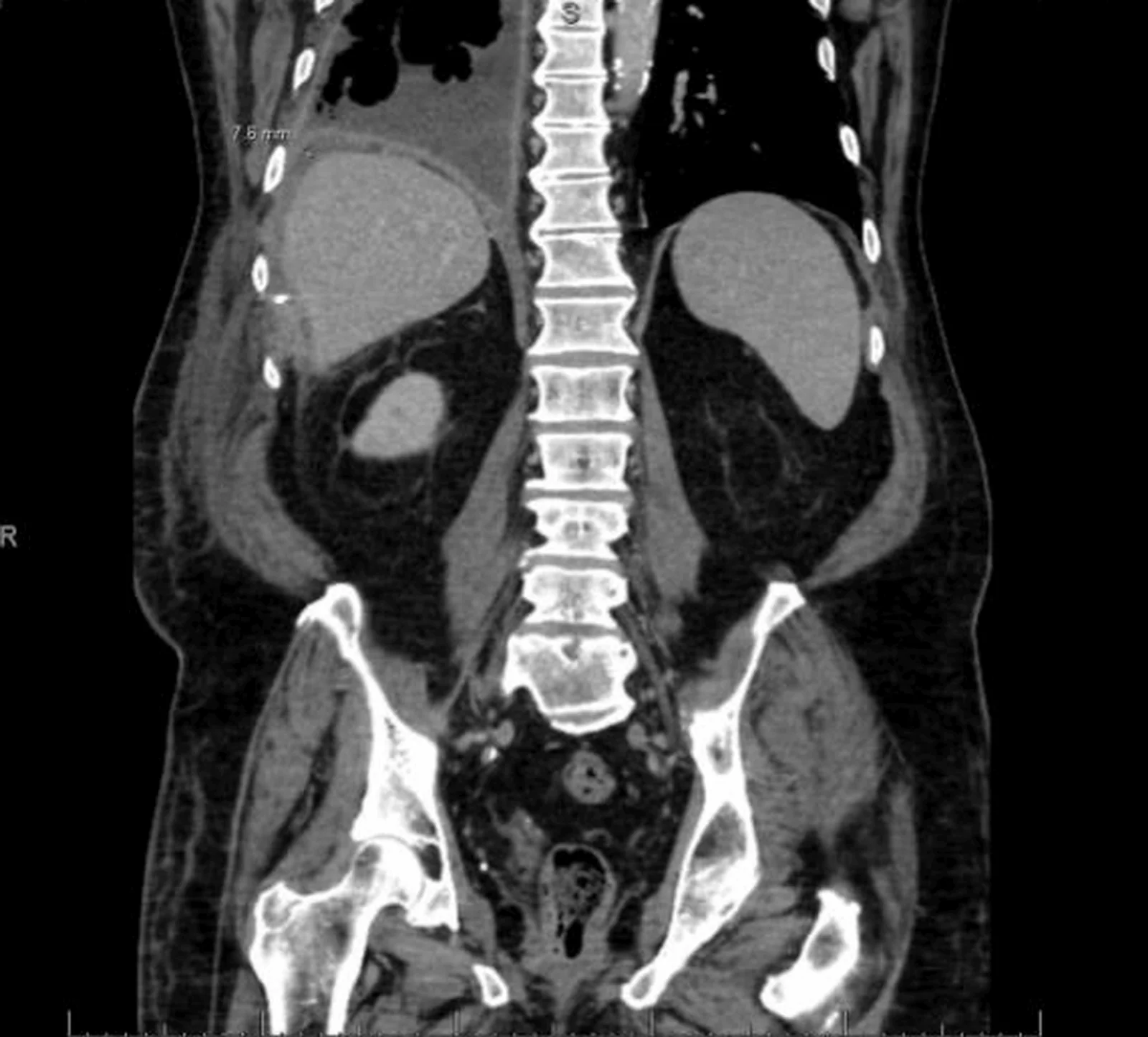
Follow-up CT of the abdomen after percutaneous drainage Coronal CT image demonstrating an interval decrease in the size of the hepatic abscess following interventional radiology-guided percutaneous drainage. The drainage catheter is visualized within the abscess cavity. CT: computed tomography

Pleural fluid analysis revealed purulent fluid. Gram stain demonstrated gram-negative bacilli and gram-positive cocci in clusters. Pleural fluid cultures subsequently grew *Bacteroides fragilis* and *Staphylococcus warneri*. Culture of the hepatic abscess drainage similarly yielded *Bacteroides fragilis* along with gram-positive cocci, consistent with a shared intra-abdominal source of infection. Blood cultures remained negative throughout hospitalization. The patient was initially started on empiric broad-spectrum intravenous antibiotic therapy with piperacillin-tazobactam and vancomycin to provide coverage for gram-positive, gram-negative, and anaerobic organisms. Following culture results and clinical response, antibiotic therapy was narrowed to intravenous ampicillin-sulbactam.

Despite percutaneous drainage of the hepatic abscess and continued antimicrobial therapy, the patient remained intermittently febrile with persistent signs of systemic infection. Repeat imaging demonstrated partial improvement of the pleural effusion but revealed residual loculated pleural collections, consistent with persistent empyema. Given the lack of clinical improvement, thoracic surgery was consulted, and the patient subsequently underwent video-assisted thoracoscopic surgery (VATS) with decortication for definitive management of the empyema. Two right-sided chest tubes were placed intraoperatively for postoperative drainage and were removed after several days once output decreased and imaging demonstrated improvement. Following surgical intervention, the patient demonstrated gradual clinical improvement with resolution of sepsis and improvement in respiratory status. Postoperative chest radiography demonstrated significant re-expansion of the right lung with interval improvement of the pleural effusion (Figure 6).

**Figure 6:**
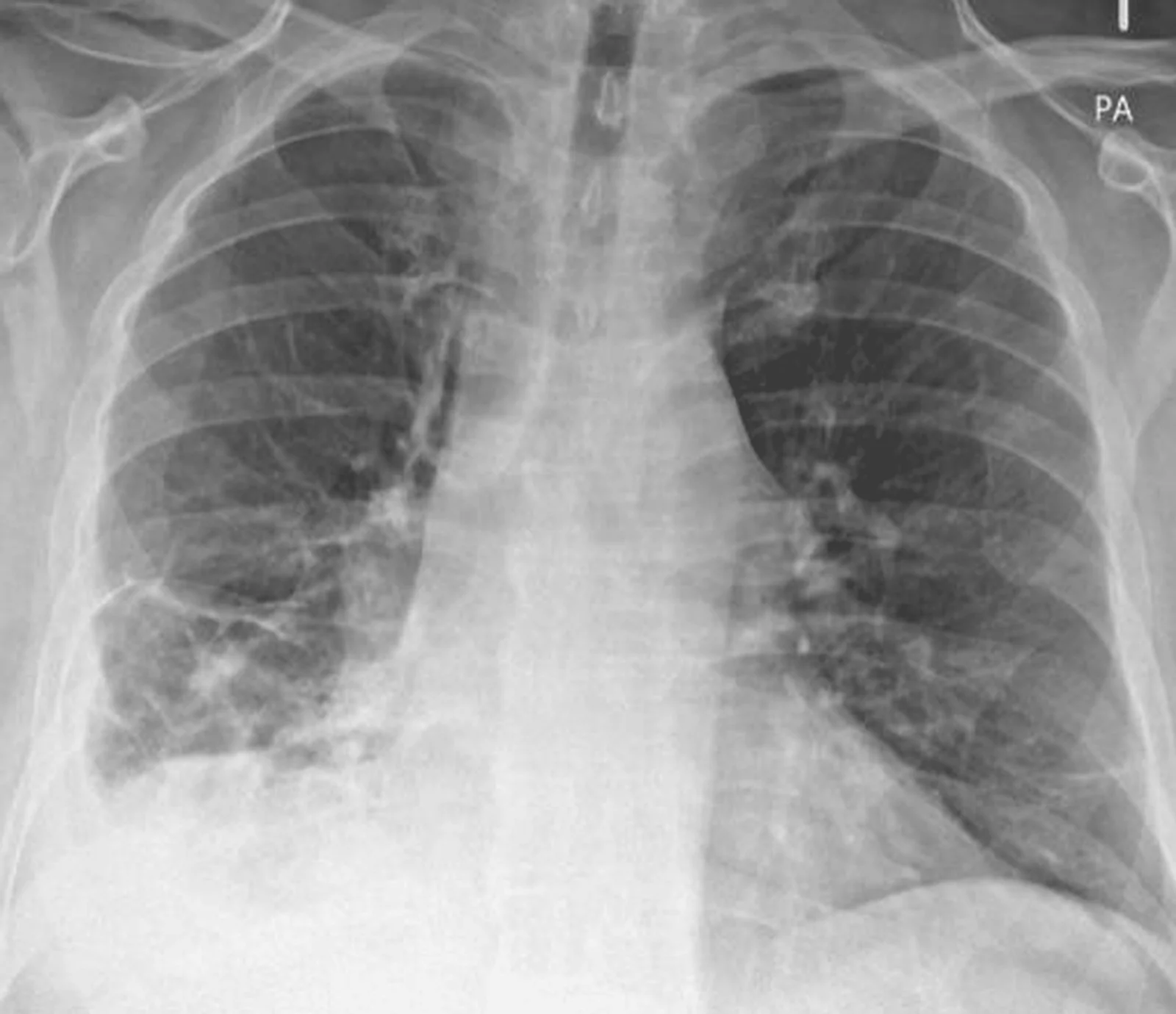
Chest radiograph after VATS decortication Follow-up chest radiograph demonstrating improved aeration and re-expansion of the right lung following VATS with decortication and removal of the pleural infection. VATS: video-assisted thoracoscopic surgery

The patient was transitioned to oral amoxicillin-clavulanate upon discharge to complete a total of four weeks of antibiotic therapy following definitive source control with surgical decortication. At outpatient follow-up with infectious disease specialists, repeat chest radiography demonstrated continued improvement of the right-sided pleural effusion, and the patient remained clinically stable without recurrence of symptoms.

## Discussion

Pyogenic liver abscess is an uncommon but potentially life-threatening infection that can result in a wide spectrum of complications depending on the extent of infection and host comorbidities. Transdiaphragmatic extension of pyogenic liver abscess into the pleural space is a rare but potentially serious complication [6]. Diabetes mellitus has been identified as an important risk factor for pyogenic liver abscess and is associated with more severe disease and increased risk of metastatic infection [7]. Gas-forming pyogenic liver abscesses have also been reported more frequently among patients with diabetes mellitus [8].

The diaphragm normally acts as an anatomical barrier separating the abdominal and thoracic cavities. However, hepatic abscesses located in the right lobe of the liver are anatomically adjacent to the diaphragm and may extend superiorly as inflammation progresses. In rare cases, rupture of the abscess or inflammatory erosion of the diaphragm may allow infection to extend directly into the pleural space [6]. This transdiaphragmatic spread may occur through direct extension or formation of a hepatopleural fistula [6]. Hematogenous spread is another potential mechanism of thoracic infection but is less consistent with the findings in our patient, given the isolation of *Bacteroides fragilis* from both the hepatic abscess and pleural fluid and the absence of bacteremia. Once bacteria gain access to the pleural cavity, they may proliferate and lead to empyema and systemic infection [6].

Anaerobic organisms such as *Bacteroides fragilis* play an important role in intra-abdominal infections. *Bacteroides fragilis* possesses virulence factors, including capsular polysaccharides, that contribute to abscess formation and modulation of host immune responses [9]. The organism is also associated with antimicrobial resistance mechanisms, including β-lactamase production [10]. Although *Bacteroides fragilis* is frequently associated with intra-abdominal infections, its presence as a pathogen in empyema is less commonly reported [1,11]. When anaerobic organisms are identified in pleural fluid, clinicians should consider aspiration, pulmonary abscess, or another contiguous source of infection, including an intra-abdominal source [11].

Pleural infection secondary to pyogenic liver abscess is an uncommon but clinically significant complication of hepatic infection [6]. Right-sided hepatic abscesses are particularly relevant because of their close anatomical relationship with the diaphragm [6]. Management requires treatment of both the hepatic and pleural sources, with antimicrobial therapy and drainage serving as the mainstays of treatment [12,13]. Contrast-enhanced CT imaging can assist in identifying hepatic abscesses and evaluating associated complications [11]. Persistent or loculated empyema despite drainage may require surgical intervention, including video-assisted thoracoscopic surgery with decortication [14,15].

Management of empyema secondary to hepatic abscess requires prompt source control combined with appropriate antimicrobial therapy [12,13]. Percutaneous drainage of the hepatic abscess is an established approach to source control and has reduced the need for open surgical drainage in appropriately selected patients [12]. Simultaneously, pleural drainage through thoracentesis or chest tube placement is often necessary to evacuate infected pleural fluid [14]. Empiric antimicrobial therapy should provide coverage against gram-negative organisms, gram-positive organisms, and anaerobic bacteria when an anaerobic pulmonary infection is suspected, particularly in cases of empyema, lung abscess, aspiration-related infection, or significant periodontal disease [11,13]. In the setting of suspected infection arising from an intra-abdominal source, such as a hepatic abscess, coverage for anaerobic organisms including *Bacteroides* species should also be considered [13].

In cases where empyema becomes loculated or fails to respond to drainage procedures, surgical intervention may be required [14,15]. Video-assisted thoracoscopic surgery with decortication is an important therapeutic option for complicated empyema because it facilitates removal of infected pleural material and treatment of the organized pleural space [14,15]. Early surgical consultation should be considered when imaging demonstrates persistent loculations or when clinical improvement is not observed despite appropriate drainage and antimicrobial therapy [14,15].

This case highlights several important clinical considerations. Clinicians should maintain a high index of suspicion for intra-abdominal sources when evaluating empyema, particularly when anaerobic organisms are identified in pleural fluid cultures [11]. Right-sided pleural effusions occurring in the setting of abdominal symptoms or systemic infection should prompt evaluation for hepatic pathology, including liver abscess [6,11]. Early multidisciplinary collaboration involving interventional radiology, thoracic surgery, and infectious disease specialists is important when both abdominal and pleural infections are present. Prompt recognition and coordinated source control are essential in the management of these complex infections [12,14].

## Conclusions

Empyema secondary to rupture of a pyogenic liver abscess is a rare but potentially life-threatening complication. Identification of anaerobic organisms such as *Bacteroides fragilis* in pleural fluid should prompt evaluation for intra-abdominal infection, particularly hepatic abscess. Early diagnosis through imaging, appropriate antimicrobial therapy, and timely source control through drainage or surgical intervention are critical for successful management. Multidisciplinary care plays a central role in improving outcomes in these complex infections.
